# Three new species of *Begonia* sect. *Baryandra* from Panay Island, Philippines

**DOI:** 10.1186/s40529-017-0182-x

**Published:** 2017-06-29

**Authors:** Ching-I Peng, Rosario Rivera Rubite, Che-Wei Lin, Mark Hughes, Yoshiko Kono, Kuo-Fang Chung

**Affiliations:** 1 Research Museum and Herbarium (HAST), Biodiversity Research Center, Academia Sinica, Nangang, Taipei, 11529 Taiwan; 2 Department of Biology, College of Arts and Sciences, University of the Philippines Manila, Padre Faura, 1000 Manila, Philippines; 3 Herbarium of Taiwan Forestry Research Institute (TAIF), Taiwan Forestry Research Institute, Taipei, 10066 Taiwan; 4 Royal Botanic Garden Edinburgh, 20A Inverleith Row, Edinburgh, EH3 5LR Scotland UK; 5 The Community Center for the Advancement of Education and Research at the University of Kochi, 5-15 Eikokuji-cho, Kochi, 780-8515 Japan

**Keywords:** Conservation, Taxonomy, Limestone, Endemic, Herbarium, Phylogenetics

## Abstract

**Background:**

The flora of Panay Island is under-collected compared with the other islands of the Philippines. In a joint expedition to the island, botanists from Taiwan and the Philippines found three unknown *Begonia* species and compared them with potentially allied species.

**Results:**

The three species are clearly assignable to *Begonia* sect. *Baryandra* which is largely endemic to the Philippines. Studies of literature, herbarium specimens, and living plants support the recognition of the three new species: *Begonia culasiensis*, *B. merrilliana*, and *B. sykakiengii.* Somatic chromosomes at metaphase were determined to be 2*n* = 30 for *B. culasiensis* and 2*n* = 28 for both *B. merrilliana* and *B. sykakiengii*, congruent with those of most species in sect. *Baryandra*. Molecular phylogenetic evidence is consistent with *B. culasiensis* being a relict from the late Miocene and *B. merrilliana* and *B. sykakiengii* being younger species of Pleistocene origin.

**Conclusion:**

The continuing discovery of endemic Philippine species means the remaining fragments of both primary and secondary native vegetation in the archipelago are of increasing value in terms of natural capital. A secure future for the species could be realized through ex situ conservation collections and raising awareness with community groups.

## Background

In continuation of our taxonomic and evolutionary studies of Philippine *Begonia* (Nakamura et al. 2013; Rubite et al. 2013, 2014, 2015; Hughes et al. 2015; Tandang et al. 2016; Peng et al. 2017), we document novelties of *Begonia* on Panay, the sixth largest island of the Philippine Archipelago. Elmer Merrill was the first to explore *Begonia* in Panay Island, describing six new species, *B. collisiae* Merr., *B. lancilimba* Merr., *B. obtusifolia* Merr., *B. panayensis* Merr., *B. rubrifolia* Merr., and *B. serpens* Merr. (Merrill 1919). There were no further reports on the begonias of Panay since then (Rubite and Madulid 2010). After securing the necessary permits, a joint expedition to Panay was conducted by botanists from the University of the Philippines Manila, West Visayas State University, and Biodiversity Research Center, Academia Sinica. The group visited four provinces of Panay and found three new species of *Begonia*. From the Municipality of Culasi, Province of Antique are two distinct species: *Begonia culasiensis* C.I Peng, Rubite, C.W.Lin, & K.F.Chung from Kipot Falls, Barangay Buenavista, and *Begonia merrilliana* C.I Peng, Rubite, C.W.Lin, & K.F.Chung from the foothills of Mt. Madia-as, Barangay Flores. The third new species is *Begonia sykakiengii* Rubite, C.I Peng, C.W.Lin & K.F.Chung from Igang Cave, Barangay Tapulang, Municipality of Maayon, Province of Capiz. All three new species belong to sect. *Baryandra*, conforming to the morphological delimitation of the section (Rubite et al. 2013) and previous phylogenetic placement (Hughes et al. 2015).

## Methods

### Morphology

Morphological studies were initially made in the wild where the plants were first found. Further morphological investigations were made in the laboratory based on literature, herbarium specimens, and flowering/fruiting plants cultivated in the experimental greenhouse, Biodiversity Research Center, Academia Sinica. The three new species were compared with the most closely allied members of sect. *Baryandra. Begonia culasiensis* was compared to *B. rubrifolia*; *B. merrilliana* to *B. obtusifolia*; and *B. sykakiengii* to *B. nigritarum*. Voucher specimens (*B. culasiensis*, *Peng 23759*; *B. merrilliana*, *Peng 23765* and *B. sykakiengii*, *Peng 24588*) are deposited at PNH and HAST.

### Chromosome preparation

Somatic chromosomes of *B. culasiensis*, *B. merrilliana*, and *B. sykakiengii* were examined using root tips from plants of the type collection. The methods of pretreatment, fixation and staining for chromosome observations followed Peng et al. (2015). Classification of the chromosome complements based on centromere position at mitotic metaphase follows Levan et al. (1964). Voucher specimens (*Peng 23794*, *Peng 23765*, *Peng 23890*) are deposited in HAST.

### Molecular phylogenetics

A time-calibrated molecular phylogenetic tree based on chloroplast DNA sequence data was taken from Hughes et al. (2015). The phylogeny samples 40 species of *Begonia* sect. *Baryandra* from the Philippines and places all three new species in a broad phylogenetic context, beginning with the diversification of the section in the late Miocene. Methods and voucher information for all taxa are as in Hughes et al. (2015).

## Results

### Species descriptions

#### 1. *Begonia culasiensis* C.I Peng, Rubite, C.W.Lin & K.F.Chung, sp. nov. (Figs. 1 and 2)

*Type:* Philippines. Panay Island. Province of Antique, Municipality of Culasi, Barangay Buenavista, Kipot Falls, on mossy limestone rock or soil, shaded in disturbed broadleaf forest, elevation ca. 50 m, 11°26′16′′N, 122°4′50′′E, 18 June 2012, *Ching*-*I Peng 23759*, with Kuo-Fang Chung, Chien-I Huang and Rosario Rubite (holotype PNH, isotype HAST).

**Fig. 1:**
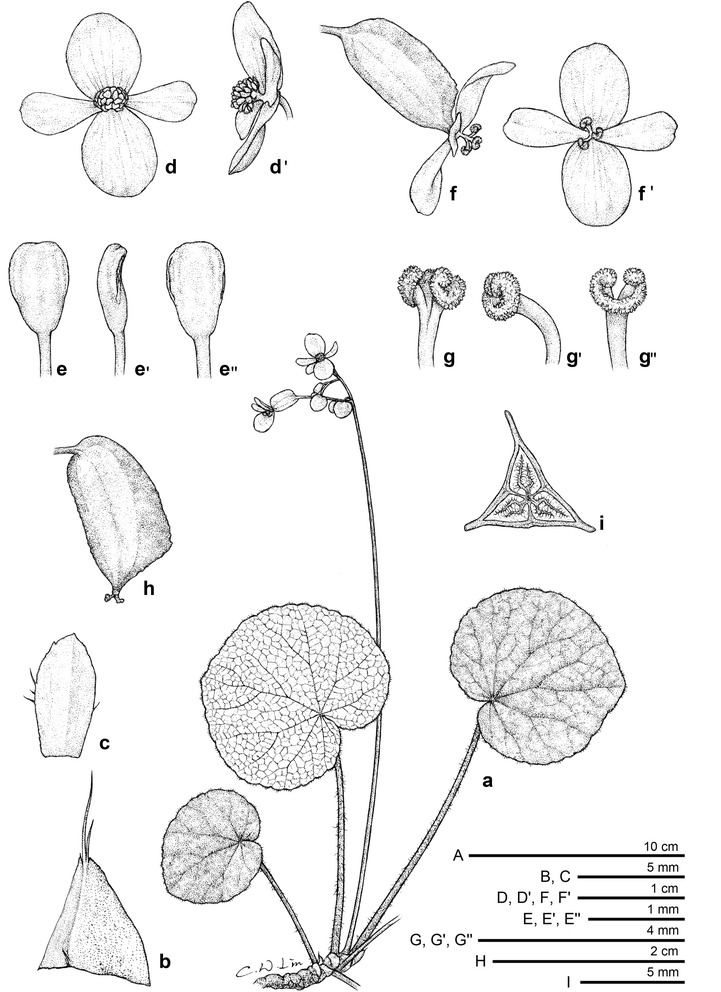
*Begonia culasiensis* C.-I Peng, R. Rubite, C.W. Lin & K.F. Chung. *A* Habit; *B* stipule; *C* Bract; *D*, *D′* staminate flower, face and side views; *E*, *E′*, *E′′* stamen, dorsal, side and ventral views; *F*, *F′* pistillate flower, face and side views; *G*, *G′*, *G′′* style and stigmatic band, ventral, side and dorsal views; *H′* capsule; *I′* cross section of an immature capsule

**Fig. 2:**
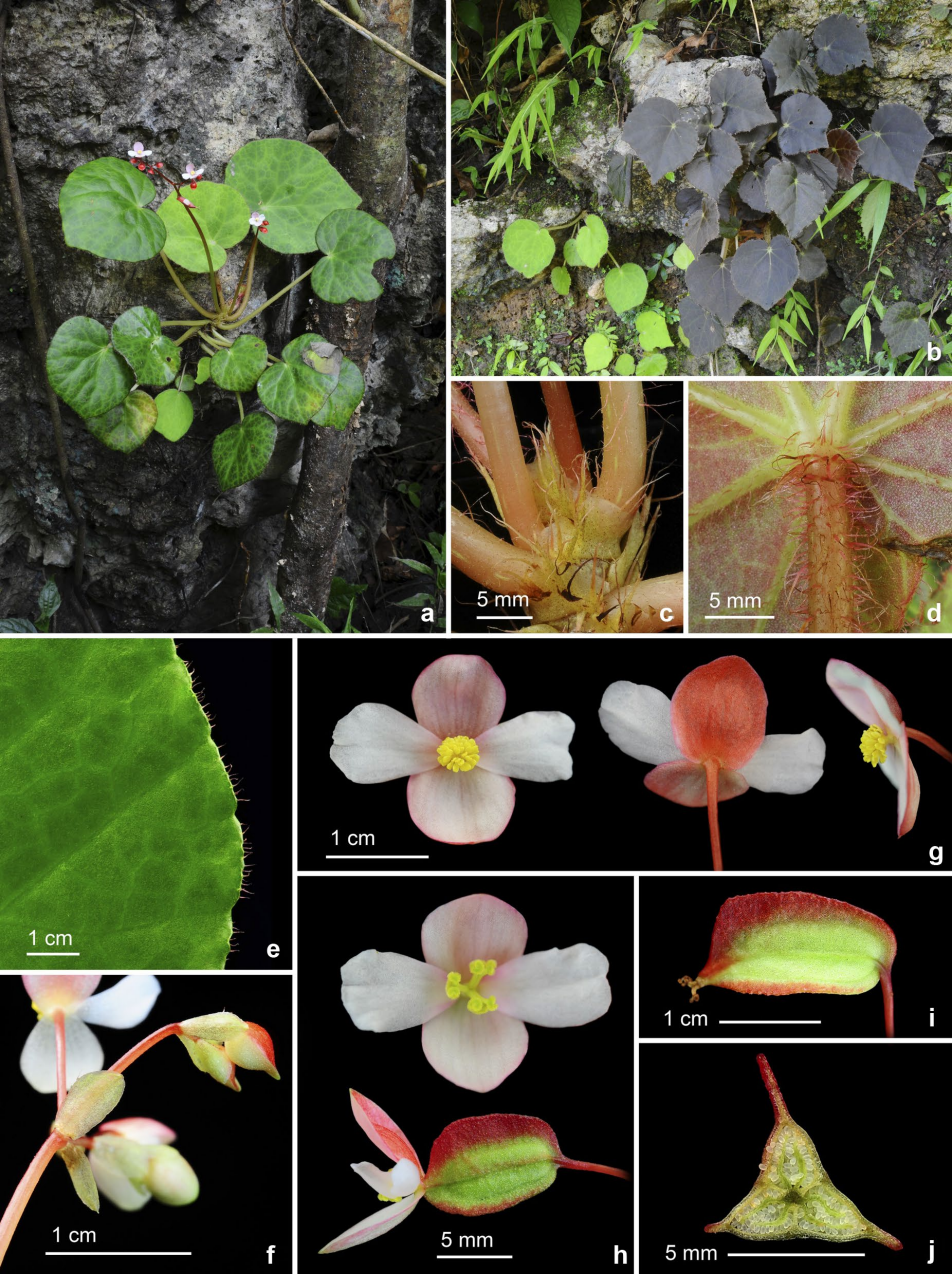
*Begonia culasiensis* C.-I Peng, R. Rubite, C.W. Lin & K.F. Chung. **a**, **b** Habitats and habits, showing leaves of varied colors; **c** rhizome, showing stipules and petiole base; **d** distal part of petiole; **e** portion of leaf, showing ciliate margin; **f** Inflorescence, showing bracts; **g** staminate flowers; **h** pistillate flowers; **i** capsules; **j** cross section of an immature capsule

Monoecious rhizomatous herb. Rhizome creeping, 10 cm or longer, 5–8 mm thick, internodes congested. Stipules persistent, pale yellowish-green, widely or very widely triangular, 5–8 mm long, 7–12 mm wide, herbaceous, strongly keeled, very sparsely velutinous on outside midrib or glabrous, apex aristate, arista 5–8 mm long. Leaves alternate, petiole terete, pale yellowish-green to reddish, 6.5–19 cm long, 3.5–6 mm thick, sparsely white or pale magenta velutinous, densely hirsute at the joint with leaf blade, fleshy hairs fused into a ring at petiole base; leaf blade asymmetric, oblique, widely ovate to suborbicular, 7.5–14.5 cm long, 5.8–13 cm wide, broad side 3.5–7.7 cm wide, basal lobes cordate, 2–3.5 cm long, apex obtuse to acuminate, sometimes rounded; margin denticulate, ciliate, hairs white or pale magenta, leaves thickly chartaceous to succulent, adaxially green or maroon to greyish–blackish, sometimes with green venation against darker background; surface glabrous; abaxially pale green or reddish, sparsely velutinous on all veins; venation palmate with ca. 7 veins, midrib distinct, with ca. 2 secondary veins on each side, other primary veins branching dichotomously or nearly so, tertiary veins reticulate. Inflorescence an axillary, bisexual and protandrous, dichasial cyme 17–39 cm long, peduncle 11–25 cm long, arising directly from rhizome, branched 5 or 6 times, erect or ascending, yellowish-green to pinkish, glabrous or subglabrous, with sparse sessile, minute glands on upper part. Bracts pale yellowish-green or reddish, hyaline, deciduous, those at basal node of inflorescence ovate, boat-shaped, 6–9 mm long, 4.5–6 mm wide, apex obtuse or acute, margin entire or repand, sparsely velutinous; bracts at the distal part of inflorescence ovate, to ca. 2 mm long, 1.5 mm wide. Staminate flower: pedicel 0.8–2 cm long, with sessile glands, tepals 4, white to pinkish, outer 2 scarlet red to pinkish outside, widely ovate or obovate to suborbicular, 0.8–1.4 cm long, 0.5–1 cm wide, outside glabrous or with sparse sessile glands; inner 2 obovate or oblanceolate, 0.8–1.2 cm long, 0.4–0.8 cm wide, apex rounded to retuse; androecium actinomorphic, ca. 0.4 cm across; stamens yellow, 27–47; filaments shortly fused at base; anthers obovate, ca. 0.8 mm long, apex truncate to retuse, subequal to filaments. Pistillate flower: pedicel 1.4–3 cm long, glabrous or with sparse sessile glands; ovary green, reddish, body trigonous-oblongoid, 5–10 mm long, 2–3.5 mm thick (wings excluded), glabrous; 3-winged, wings 7–12 mm long, lateral wings narrower, narrowly linear, 0.5–1.2 mm wide, abaxial wing much protruded, narrowly linear or oblanceolate, attenuate on both ends, 1–2.5 mm wide, margin entire; ovary 3-locular, placenta bilamellate; tepals 4, white to pinkish, outer 2 obovate, widely ovate or widely obovate, 9–1.1 cm long, 0.7–8.5 cm wide, with sparse sessile glands or glabrous outside; inner 2 oblanceolate to widely obovate, 0.7–0.9 cm long, 0.3–0.6 cm wide, apex truncate to retuse; styles 3, shortly fused at base, yellow, 2.5–4 mm long, stigma spirally twisted. Capsule pendent, pedicel 1.4–3.2 cm long, tepals deciduous; body trigonous-oblongoid, 9–14 mm long, 4–6 mm across (wings excluded), greenish or reddish when fresh; wings unequal, ca. 15–19 mm long; lateral wings ca. 2 mm wide, abaxial wing 3–4 mm wide. Somatic chromosome number, 2*n* = 30.

*Additional specimens examined:* Philippines. Panay Island. Province of Antique, Municipality of Culasi, Barangay Alojipan, Guintungaban, by riverbanks, rock face, shaded by a lot of trees, ca. 60 m alt. 18 Mar 2006, *Rosario Rubite 234* (HAST) (plants of this collection were brought back for cultivation. This specimen was pressed from greenhouse material on 1 Feb 2007); Province of Antique, Municipality of Culasi, Barangay, Camancijan. Disturbed broadleaf forest. ca. 75 m alt., 11°27′19′′N, 122°5′9′′E. 18 June 2012, leaves green. *Ching*-*I Peng 23793* with Kuo-Fang Chung, Chien-I Huang and Rosario Rubite (HAST); *loc. cit*., leaves red. *Ching*-*I Peng 23794* with Kuo-Fang Chung, Chien-I Huang and Rosario Rubite (HAST).

*Distribution and ecology:* *Begonia culasiensis* is endemic to Culasi Municipality, Panay Island. It grows on semishaded rocky slopes in evergreen forests at 50–75 m in elevation.

*Etymology:* Named for its locality, the Municipality of Culasi, where the new species was discovered.

*Notes:* *Begonia culasiensis* resembles *B. rubrifolia*, also from western Panay, in having short rhizomes and widely ovate leaves. However, *B. culasiensis* can be distinguished from *B. rubrifolia* by a combination of characters, including the larger lamina (7.5–14.5 × 5.8–13 vs. 6–9 × 8–10 cm), longer petiole (6.5–19 vs. 7–11 cm), lesser stamens (27–47 vs. 50–60), shorter anthers (ca. 0.8 mm vs. 1–2 mm), and narrower (oblong vs. rhomboidal to orbicular) capsules. Indeed, *Begonia culasiensis* is notable in being the only Philippine *Begonia* with oblongoid capsules (i.e., Figs. 1, 2). Ecologically, *B. culasiensis* occurs in lowland forest at ca. 50 m in elevation, whereas *B. rubrifolia* grows in montane forest at ca. 1000 m in elevation.

*Provisional IUCN category:* Following the Red List criteria of the IUCN (2012), we consider *Begonia culasiensis* to belong to the Vulnerable category under criterion D2 (population with a very restricted area of occupancy). Although there are several sub-populations along the forest trail in the Municipality of Culasi where the species was discovered, heavy use of this trail has led to an observed decline in numbers between visits by one of the authors in 2006 and 2012.

#### 2. *Begonia merrilliana* C.I Peng, Rubite, C.W.Lin & K.F.Chung, sp. nov. (Figs. 3 and 4)

*Type:* Philippines. Panay Island, Antique Province, Culasi Municipality, Barangay Flores. At stream bank on soil slope, semi-exposed, wet, elevation ca. 225 m, 11°22′11′′N, 122°6′33′′E, 19 June 2012. *Ching*-*I Peng 23765*, with Kuo-Fang Chung, Chien-I Huang and Rosario Rubite (holotype PNH, isotype HAST).

**Fig. 3:**
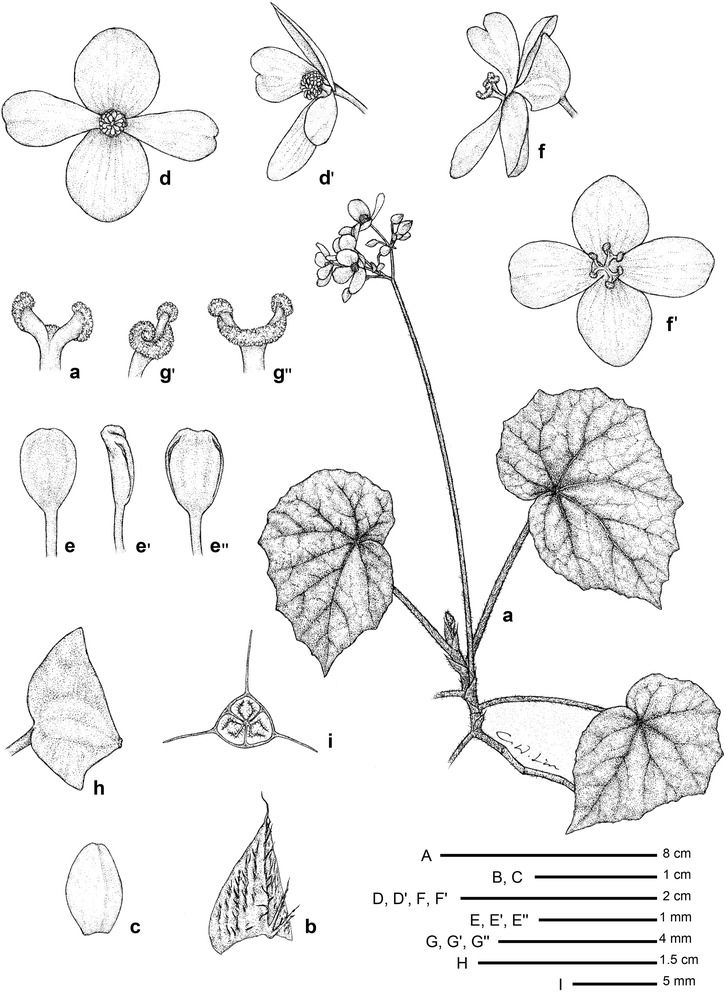
*Begonia merrilliana* C.-I Peng, R. Rubite, C.W. Lin & K.F. Chung*. A* Habit; *B* stipule; *C* bract; *D*, *D′* staminate flower, face and back views; *E*, *E*′, *E*′′ stamen, dorsal, side and ventral views; *F*, *F*′ pistillate flower, side and face views; *G*, *G*′, *G*′′ style and stigmatic band, ventral, side and dorsal views; *H* Capsule; *I* Cross section of an immature capsule

**Fig. 4:**
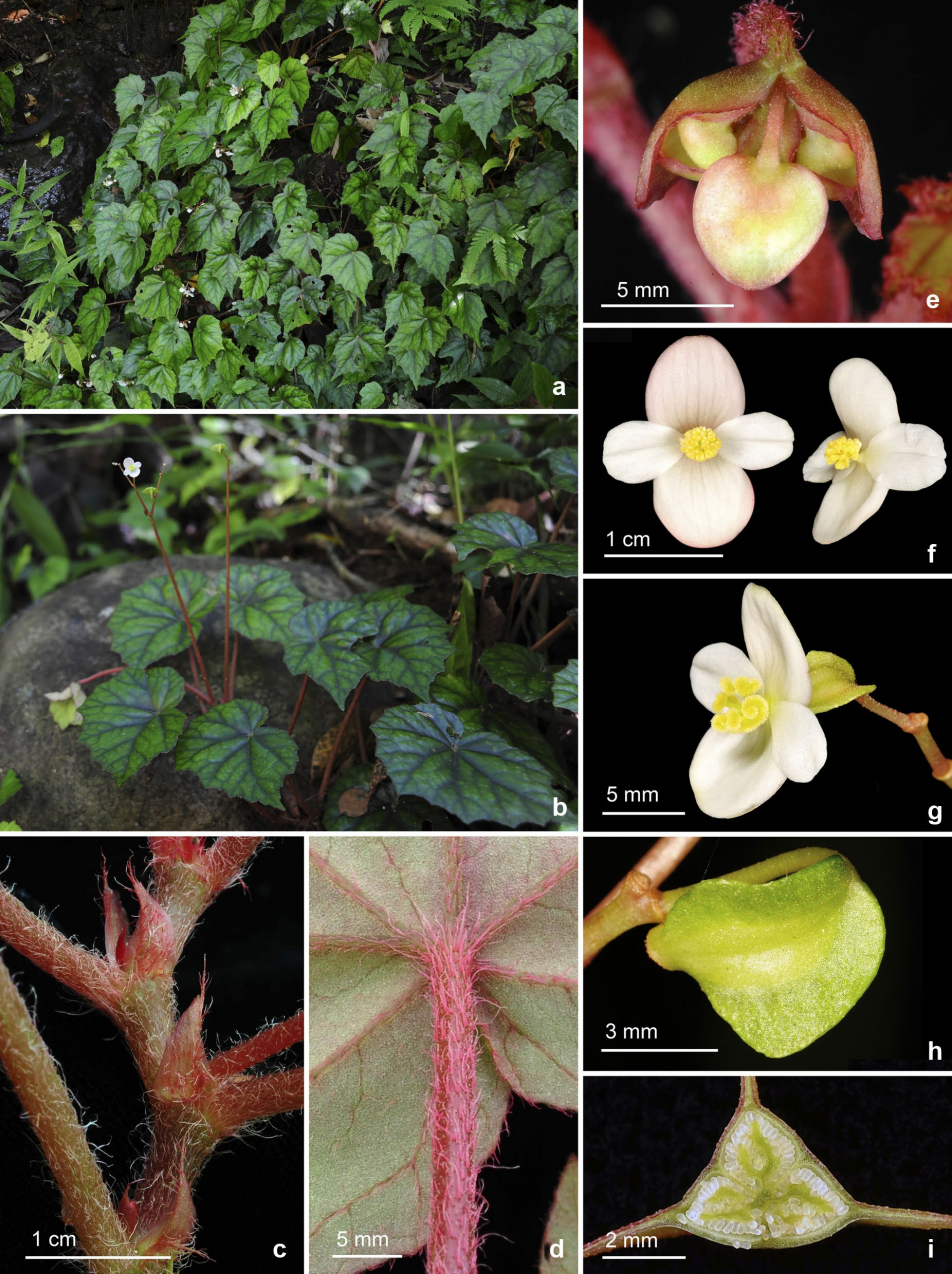
*Begonia merrilliana* C.-I Peng, R. Rubite, C.W. Lin & K.F. Chung. **a**, **b** Habitat and habit; **c** rhizome, showing stipules and petioles; **d** distal part of petiole, showing appressed villous vestiture; **e** immature inflorescence, showing bracts; **f** staminate flowers; **g** pistillate flower; **h** capsule; **i** cross section of an immature capsule

Monoecious rhizomatous herb. Rhizome pale olive green to reddish, long creeping, to 30 cm or longer, 0.4–0.6 cm thick, internodes 0.8–5 cm long, densely appressed pink villous. Stipules persistent, pale yellowish-green or reddish, triangular or narrowly triangular, 8–14 mm long, 3–8 mm wide, herbaceous, strongly keeled, sparsely velutinous, margin entire, apex aristate, arista 3.5–6 mm long. Leaves alternate, distant, petiole terete, pale red, (3.5–) 9–14 cm long, 3.5–5 mm thick, densely appressed pink villous; leaf blade asymmetric, oblique, ovate, 7.5–12.5 cm long, 4–8 cm wide, broad side 3.5–5.8 cm wide, basal lobes cordate, 2–5 cm long, apex attenuate to caudate, margin irregularly angular and undulate, ciliate, hairs pinkish; leaves thickly chartaceous to succulent, adaxially green, dark green or with a brown band along veins, sometimes variegated with grayish-green spots between major veins; surface very sparsely velutinous or subglabrous; abaxially pale green or reddish, pinkish velutinous on all veins; venation palmate with ca. 7 veins, midrib distinct, with ca. 2 secondary veins on each side, other primary veins branching dichotomously, tertiary veins reticulate. Inflorescences an axillary, bisexual and protandrous, dichasial cyme 8–23 cm long, peduncle 6.5–17 cm long, dichasial cymes arising directly from rhizome, branched ca. 5 times, erect or ascending, pinkish, reddish villous. Bracts pale yellowish-green, hyaline, deciduous, glabrous or subglabrous, those at basal node of inflorescence ovate to widely ovate, boat-shaped, ca. 8 mm long, 4 mm wide, apex obtuse, margin entire or very sparsely velutinous; bracts at distal part of inflorescence similar but smaller. Staminate flower: pedicel 1 cm long, glabrous, tepals 4, white to pinkish, glabrous, outer 2 widely ovate or obovate to suborbicular, 0.7–1.1 cm long, 0.8–1 cm wide, inner 2 obovate or oblanceolate, 0.6–1.4 cm long, 0.4–0.7 cm wide, apex truncate to retuse; androecium actinomorphic, ca. 0.3 cm across; stamens yellow, 38–62; filaments shortly fused at base; anthers obovate, ca. 0.8 mm long, apex rounded to retuse, subequal to filaments. Pistillate flower: pedicel 0.7–1.4 cm long, glabrous, with sparse glands; ovary yellowish green to reddish, body trigonous-ellipsoid, ca. 5 mm long, 3.5 mm thick (wings excluded), glabrous, with sparse sessile glands; 3-winged, subequal, wings ca. 6 mm long, triangular or so, apex obtuse to acute, 1–4 mm wide, margin entire; ovary 3-locular, placenta bilamellate; tepals 4, white to pinkish, glabrous, outer 2 obovate, widely ovate or obovate, 0.6–1 cm long, 0.6–0.9 cm wide, inner 2 oblanceolate to widely obovate, 1–1.2 cm long, 0.6–0.7 cm wide, apex truncate to retuse; styles 3, shortly fused at base, yellow, ca. 0.35 cm long, stigma spirally twisted. Capsule pendent, pedicel 1–1.9 cm long, tepals deciduous; body trigonous-ellipsoid, ca. 6 mm long, 4 mm across (wings excluded), greenish or reddish when fresh; wings subequal, ca. 7 mm long, 2–6 mm wide. Somatic chromosome number, 2*n* = 28.

*Distribution and ecology:* *Begonia merrilliana* is endemic to Panay. On rock-strewn cliff base or along stream banks in lowland forest, 200–300 m elevation.

*Etymology:* Named in honor of Dr. Elmer Drew Merrill, an outstanding plant systematist who worked in the Philippines for more than two decades in the early twentieth century.

*Notes:* *Begonia merrilliana* is similar to *B. obtusifolia* in the elongate, creeping rhizomes and undulate, angular leaf margin. However, *B. merrilliana* differs in having shorter petioles (3.5–14 cm vs. 20–30 cm) long, narrower leaves (7.5–12.5 × 4–8 cm vs. 7–13 × 6–14 cm), leaf margin irregularly angular and undulate (vs. evidently lobed), relatively flat (vs. strongly rugose) and variegated (vs. uniformly green) on the upper side; staminate outer tepals 7–11 × 8–10 mm (vs. 13–15 × 12–14 mm), stamens ca. 0.8 mm (vs. 1–1.5 mm) long; pistillate outer tepals 6–10 × 6–9 mm (vs. 10–12 × 10–12 mm).

*Provisional IUCN category:* Following the Red List criteria of the IUCN (2012), we consider *Begonia merrilliana* to belong to the vulnerable category under criterion D2 (population with a very restricted area of occupancy). The species is restricted to Antique Province on Panay, and forest cover in the region (and hence suitable habitat) is very low.

#### 3. *Begonia sykakiengii* Rubite, C.I Peng, C.W.Lin & K.F.Chung, sp. nov. (Figs. 5, 6, 7 and 8)

*Type:* Philippines. Panay Island. Capiz Province, Maayon Municipality, Tapulang Barangay, Igang Cave. On moist limestone rock or soil, semishaded, elevation ca. 15 m, 11°23′00′′N, 122°47′00′′E, 22 Jun 2012, *Ching*-*I Peng 23836* with Kuo-Fang Chung, Chien-I Huang, Rosario Rubite, Lucia L. Lastimosa and Kym Lopez (holotype PNH, isotype HAST).

**Fig. 5:**
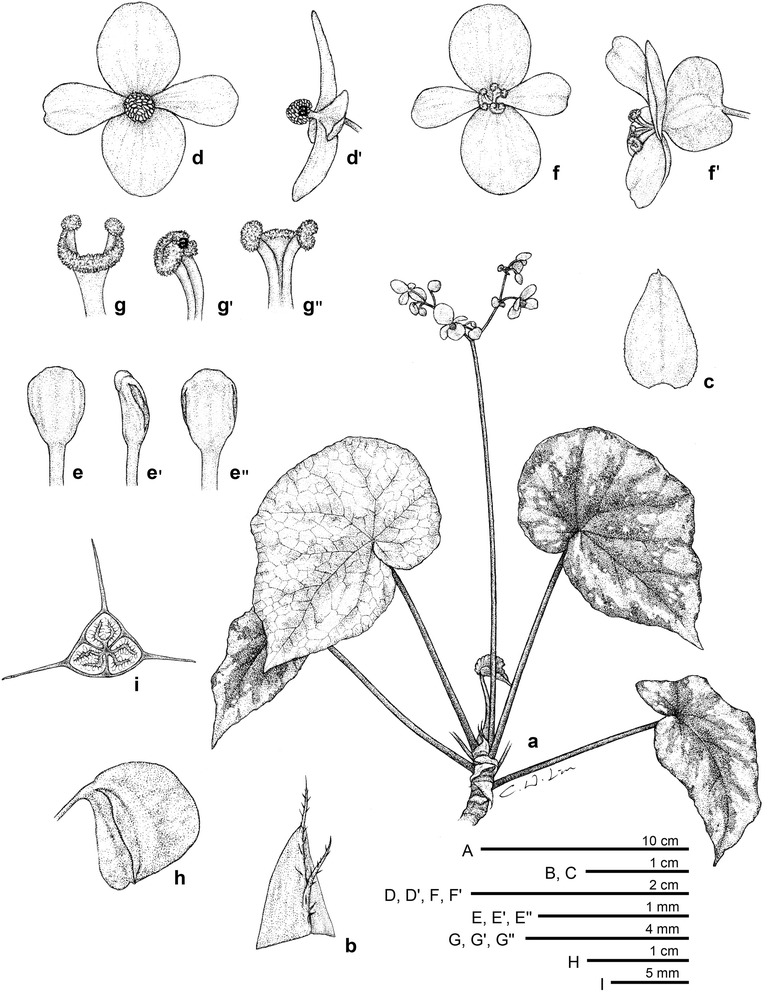
*Begonia sykakiengii* R. Rubite, C.-I Peng, C.W. Lin & K.F. Chung*. A* habit; *B* stipule; *C* bract; *D*, *D′* staminate flower, face and side views; *E*, *E*′, *E*′′ stamen, dorsal, side and ventral views; *F*, *F*′ pistillate flower, face and side views; *G*, *G*′, *G*′′ style and stigmatic band, dorsal, side and ventral views; *H* capsule; *I* cross section of an immature capsule

**Fig. 6:**
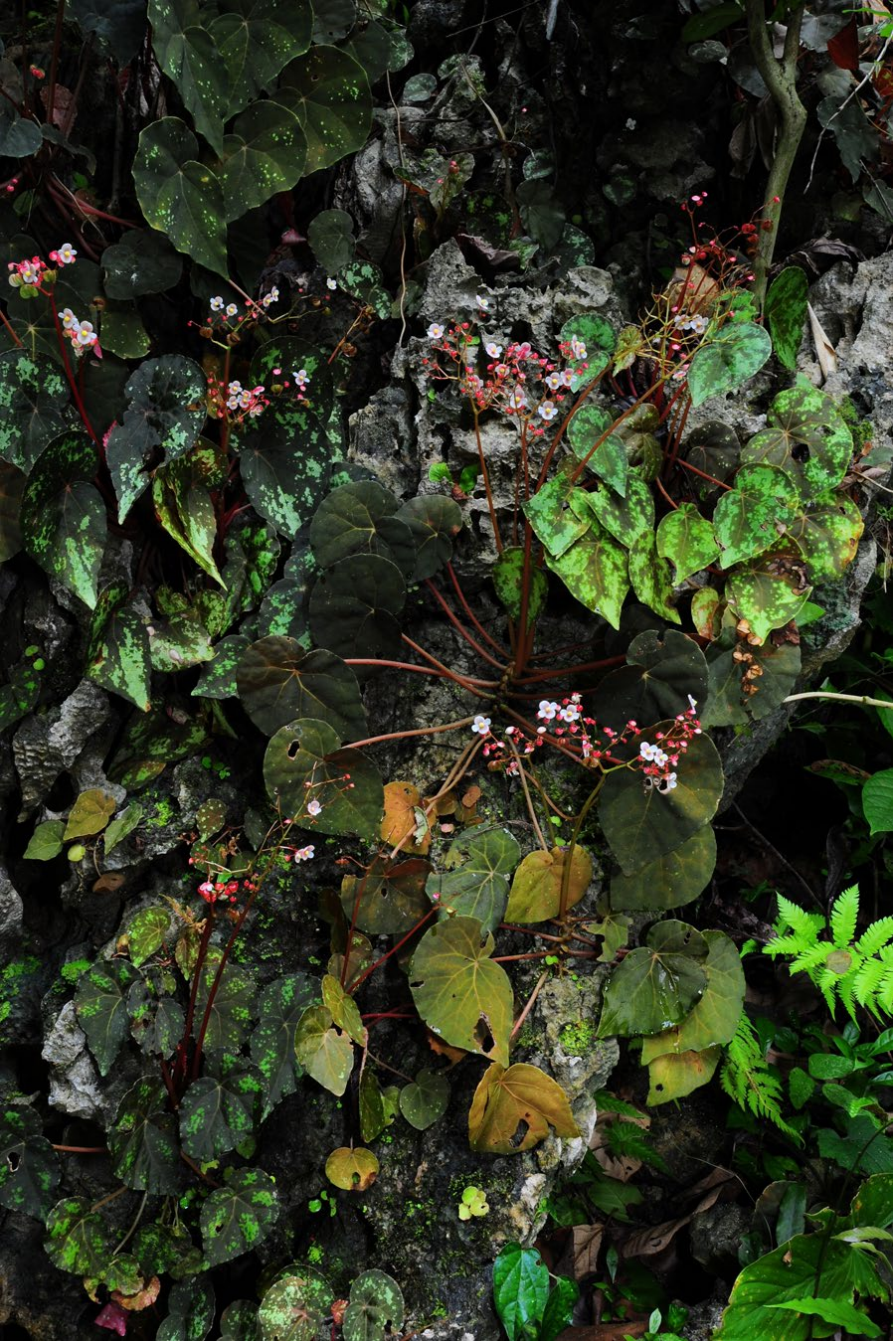
*Begonia sykakiengii* R. Rubite, C.-I Peng, C.W. Lin & K.F. Chung on limestone cliff, showing variably colorful foliage

**Fig. 7:**
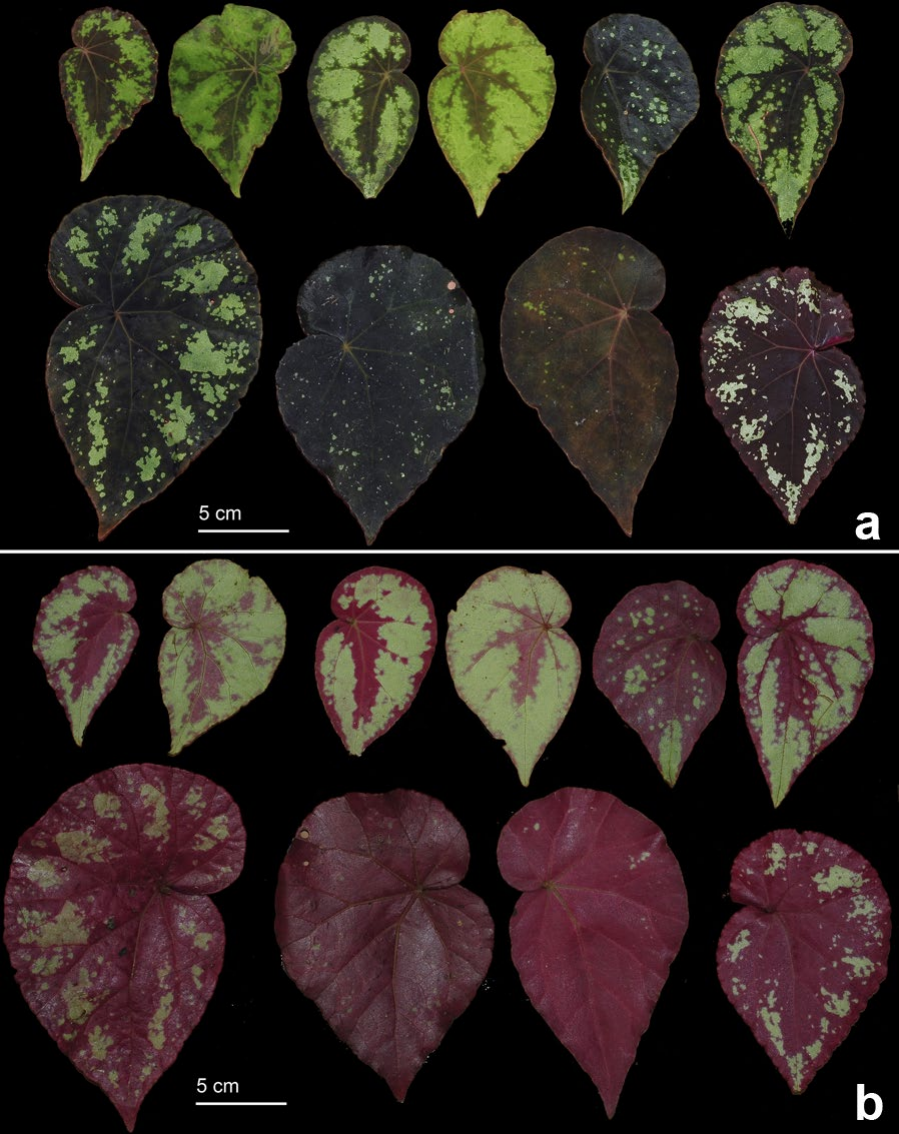
*Begonia sykakiengii* R. Rubite, C.-I Peng, C.W. Lin & K.F. Chung. Leaves photographed in the field to show varied and complex foliar maculation patterns on both surfaces. **a** Adaxial surface; **b** abaxial surface

**Fig. 8:**
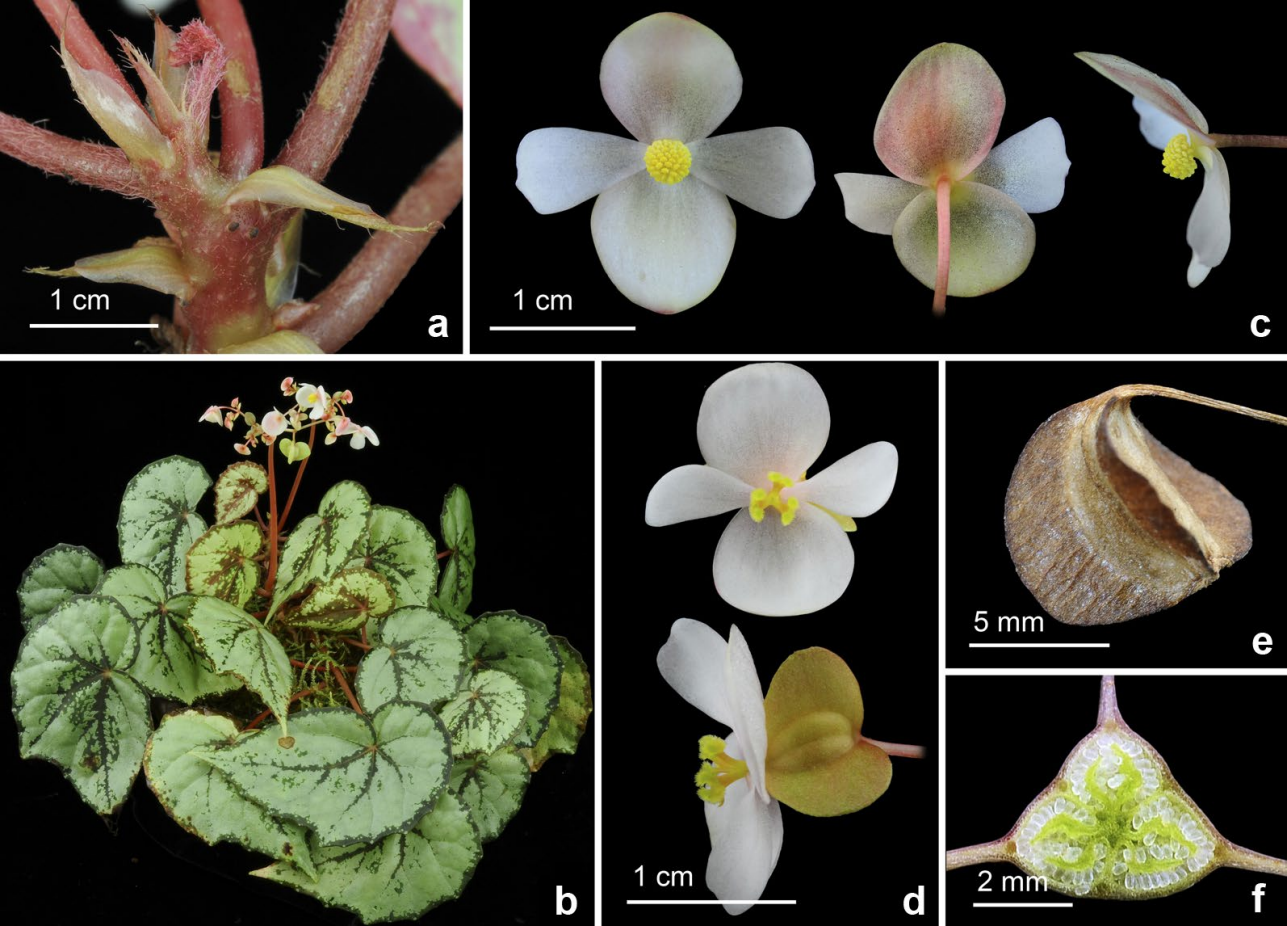
*Begonia sykakiengii* R. Rubite, C.-I Peng, C.W. Lin & K.F. Chung. **a** Apical part of a rhizome, showing stipules and petioles; **b** habit; **c** staminate flowers; **d** pistillate flowers; **e** capsule; **f** cross section of an immature capsule

Monoecious rhizomatous herb. Rhizome creeping, to 15 cm or longer, 5–12 mm thick, internodes 2–8 mm long. Stipules persistent, pale yellowish-green, triangular, 7–16 mm long, 6.5–12 mm wide, herbaceous, strongly keeled, velutinous on midrib, margin entire, apex aristate, arista 2–3 mm long. Leaves alternate, petiole terete, reddish to crimson, (3.5–) 8–24.5 cm long, 2.5–6 mm thick, densely appressed velutinous; leaf blade asymmetric, oblique, ovate to widely ovate, 8.5–18 cm long, 5–12 cm wide, broad side 3.4–9 cm wide, basal lobes cordate, 2.2–7 cm long, ape attenuate to caudate, margin undulate-denticulate or subentire (sometimes with one angular lobe), appressed velutinous; leaves thickly chartaceous to succulent, adaxially green, dark olive green to maroon or nearly blackish, variegated with irregular spots of chartreuse, silver grey to whitish, usually forming an wide band running parallel to the margin, sometimes lamina uniformly dusky green to maroon; surface glabrous; abaxially purplish red, with patches of pale green corresponding to the pattern on adaxial surface, all veins of which sparsely appressed velutinous; venation palmate with ca. 8 veins, midrib distinct, with ca. 2 secondary veins on each side, other primary veins branching dichotomously or nearly so, tertiary veins reticulate. Inflorescences an axillary, bisexual and protandrous, dichasial cyme 15–35 cm long, peduncle 9–32 cm long, dichasial cymes arising directly from rhizome, branched ca. 7 times, erect or ascending, reddish, glabrous. Bracts pale yellowish-green, hyaline, deciduous, those at basal node of inflorescence ovate, 7–11 mm long, 4–7 mm wide, apex acute, sparsely shortly glandular hairy outside, margin entire with shortly glandular hairy; bracts at the distal part of inflorescence similar but smaller. Staminate flower: pedicel 1.3–2.2 cm long, shortly glandular hairy, tepals 4, white to pinkish, sparsely shortly glandular hairy on outside, outer 2 widely ovate or obovate to suborbicular, 0.6–1.2 cm long, ca. 1 cm wide, inner 2 obovate, 0.6–1 cm long, 0.5–0.7 cm wide, apex truncate to rounded; androecium actinomorphic, ca. 0.35 cm across; stamens yellow, 53–70; filaments shortly fused at base; anthers obovate, ca. 0.7 mm long, apex rounded, subequal to filaments. Pistillate flower: pedicel 0.6–1.1 cm long, glabrous or sometimes with sparsely shortly glandular hairy; ovary pale yellowish green to reddish, body trigonous-ellipsoid, 5–6 mm long, 2.5–4 mm thick (wings excluded), glabrous, 3-winged, subequal, subtriangular or crescent-shaped, cuneate distally, rounded proximally, apex rounded or obtuse, wings 6–7.5 mm long, 6–10 mm wide, margin entire; ovary 3-locular, placenta bilamellate; tepals 4, white to pinkish, glabrous, outer 2 suborbicular to widelPhilippines. Panay Islandy obovate, 0.7–1.2 cm long, 0.9–1.2 cm wide, inner 2 oblanceolate to widely obovate, 0.9–1 cm long, 0.3–7 cm wide, apex rounded to truncate; styles 3, shortly fused at base, yellow, ca. 0.3 cm long, stigma spirally twisted. Capsule pendent, pedicel ca. 1 cm long, tepals deciduous; body trigonous-ellipsoid, ca. 6 mm long, 4 mm across (wings excluded), greenish or reddish when fresh; wings subequal, wings ca. 10 mm long, 5 mm wide. Somatic chromosome number, 2*n* = 28.

*Distribution and ecology:* *Begonia sykakiengii* is endemic to the Municipality of Maayon. On vertical cliffs face of exposed or semi-exposed limestone hills, at ca. 0–50 m in elevation.

*Etymology:* Mr. Sy Ka Kieng, patriarch of a Chinese family who has supported R. Rubite’s field trips.

*Notes:* *Begonia sykakiengii* resembles *Begonia nigritarum*, a widespread species in the Philippines, but the new species differs in having larger stipules (7–16 × 6.5–12 vs. 5–8 × 3–5 mm), larger leaf blades (8.5–18 × 5–12 vs. 4–6 × 4–5 cm), larger bracts at the base of inflorescence (7–11 × 4–7 vs. 3–4 × 2–3 cm), longer inflorescence peduncle (to 32 vs. to 6 cm), and longer styles (ca. 3 vs. 1.5–2 mm). *Begonia sykakiengii*, with its magnificent colorful foliage that are either dotted or forming patches or ring maculation on the upper side, and gorgeous purplish red beneath, may be considered one of Panay’s most handsome native species.

*Provisional IUCN category:* Following the Red List criteria of the IUCN (2012), we consider *Begonia sykakiengii* to belong to the Endangered category under criteria B2ab(iii) [(a) severely fragmented or known to exist at no more than five locations; (biii) continuing observed decline in quality of habitat). This species is a micro-endemic, restricted to a single cave which is not in a formally protected area. The site is surrounded by rice fields and habitation, and graffiti at the cave entrance indicates the site is already disturbed.

### Chromosome cytology

**Fig. 9:**
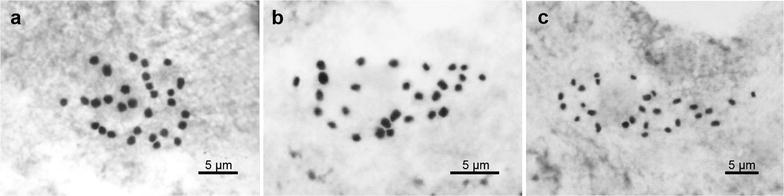
Somatic chromosomes at metaphase of *Begonia*. **a** *B. culasiensis* (2*n* = 30, *Peng* et al*. 23794*); **b** *B. merrilliana* (2*n* = 28, *Peng* et al*. 23765*); **c** *B. sykakiengii* (2*n* = 28, *Peng* et al*. 23890*)

Philippine species of *Begonia* formerly placed in sect. *Diploclinium* were transferred to sect. *Baryandra* based on molecular evidence (Rubite et al. 2013). Among 48 species recognized in sect. *Baryandra*, chromosome numbers of ten species were previously reported: *B. blancii* (2*n* = 30, Hughes et al. 2011), 2*n* = 28, Nakamura et al. 2013), *B. chingipengii* (2*n* = 28, Rubite et al. 2014), *B. fenicis* (2*n* = 26, Oginuma and Peng 2002; 2*n* = 28, Nakamura et al. 2013; 2*n* = 56, Kokubugata and Madulid 2000); *B. hughesii* (2*n* = 30, Rubite et al. 2015), *B. parva* (2*n* = 36 + 2f, Legro and Doorenbos 1969), *B. rhombicarpa* (2*n* = 44, Doorenbos et al. 1998), *B. suborbiculata* (2*n* = 30, Hughes et al. 2011), *B. tagbanua* (2*n* = 30, Rubite et al. 2015), *B. tandangii* (2*n* = 28, Nakamura et al. 2013) and *B. taraw* (2*n* = 28, Rubite et al. 2015). In our study, 2*n* = 30 was determined for *B. culasiensis* (Fig. 9a), and 2*n* = 28 was determined for *B. merrilliana* and *B. sykakiengii* (Fig. 9b, c, respectively). Our data are congruent with those of most species in sect. *Baryandra* for which chromosome numbers are known.

### Molecular phylogenetics

**Fig. 10:**
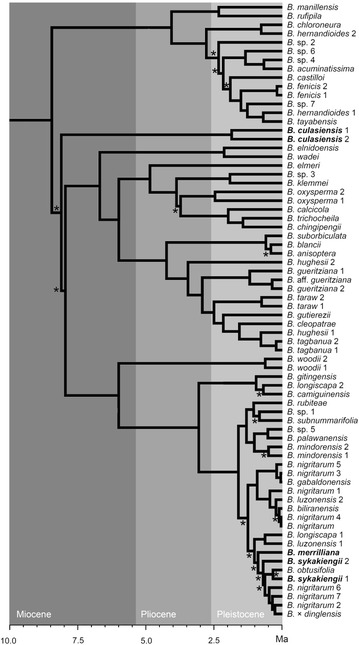
A chronogram based on chloroplast DNA sequence data, taken from Hughes et al. (2015), with the three new species highlighted in *bold font*. Geological epochs are indicated with *three shades of grey* in the background: *light*; Holocene and Pleistocene (0–2.6 Ma);* mid-grey*, Pliocene (2.6–5.3 Ma);* dark grey*, Miocene (5.3–23.0 Ma). *Asterisks* mark clades with PP < 0.95

*Begonia culasiensis* represents one of the earliest branching lineages in *Begonia* sect. *Baryandra* (Fig. 10), and likely represents a relict lineage which has been present on the island of Panay since the Miocene (Hughes et al. 2015). *Begonia sykakiengii* and *B. merrilliana* belong to the same clade as each other, highly nested within *Begonia* sect. *Baryandra* and of Pleistocene origin, and represent part of an endemic radiation on Panay. Two populations of *Begonia culasiensis* and *B. sykakiengii* were sampled in the phylogeny, showing some chloroplast sequence diversity to be present between populations of the species.

## Discussion

Of the 25 conservation priority biodiversity hotspots identified by Myers et al. (2000), the Philippines has the lowest percentage of remaining primary vegetation, at 3%. Hence the continuing discovery of endemic Philippine species means the remaining fragments of both primary and secondary native vegetation are of increasing value in terms of natural capital for the Philippines.

The molecular phylogenetic results show a relict endemic element to the *Begonia* flora of Panay (Hughes et al. 2015). Given the complex geological history of the island, which includes fragments of the Palawan microcontinental block (Hall 2002), further studies on different groups with an endemic element on Panay would be useful, as it is potentially a key area in the geological and biotic evolution of the archipelago.

## Conclusions

The discovery of three new species endemic to Panay highlights the importance of the island to the Philippines in conservation terms. The next step is to ensure the long-term future of the species described here in both ex situ collections in their native localities. This is an opportunity for botanic gardens and community conservation groups in the Philippines to achieve a high impact at low cost, as the species are relatively easily cultivated, and have small native populations which could potentially be protected in micro-reserves (Appendix).

## Appendix: *Begonia* specimens examined for morphological comparison

*Begonia culasiensis* C.-I Peng, R. Rubite, C.W. Lin & K.F. Chung. Panay Island. Province of Antique, Municipality of Culasi, Barangay Buenavista, Kipot Falls, 18 June 2012, *Peng 23759* (PNH, HAST).

*Begonia merrilliana* C.-I Peng, R. Rubite, C.W. Lin & K.F. Chung. Panay Island. Province of Antique, Municipality of Culasi, Barangay Flores, 19 June 2012, *Peng 23765* (PNH, HAST).

*Begonia nigritarum* E.G. Steudel. Luzon Island: Province of Laguna, Los Banos, Mt. Makiling, 4 June 1958, *J. Sinclair & G.E. Edano 9501* (PNH 55107).

*Begonia nigritarum* E.G. Steudel. Luzon Island: Province of Laguna, Los Banos, Mt. Makiling, 8 February 1976, *M.J.S. Sands, M.G. Price & E.J. Reynoso 3075* (PNH 169880).

*Begonia nigritarum* E.G. Steudel. Luzon Island: Province of Bataan, Lamao, Kamayan River, 6 December 1947, *G.E. Edano 311* (PNH 4176).

*Begonia nigritarum* E.G. Steudel. Luzon Island: Province of Nueva Vizcaya, Cordon, 10 June1902, *E.D. Merrill 143* (US 115387).

*Begonia nigritarum* E.G. Steudel. Luzon Island: Province of Pampanga, Mt. Pinatubo, Camp Stotsenburg, May 1927, *A.D.E. Elmer 21998* (K 325074).

*Begonia nigritarum* E.G. Steudel. Panay Island: Province of Iloilo, Municipality of Dingle, Bulabog Putian National Park, 24 June 2012, *Ching-I Peng 23855–23858* (PNH, HAST).

*Begonia obtusifolia* E.D. Merrill. Panay Island: Capiz Province, Mt. Macosolon. April-May 1918, *M. Ramos* and *G. Edano B.S. 30803* (US 115406).

*Begonia rubrifolia* E.D. Merrill. Panay Island: Antique Province, Municipality of Culasi, May-Aug 1918, *R.C. McGregor B.S. 32430* (BM).

*Begonia sykakiengii* R. Rubite, C.-I Peng, C.W. Lin & K.F. Chung. Panay Island. Province of Capiz, Municipality of Maayon, Barangay Tapulang, Igang Cave. 22 June 2012, *Peng 24588* (PNH, HAST).
